# Human Telomerase Reverse Transcriptase (hTERT) Q169 Is Essential for Telomerase Function *In Vitro* and *In Vivo*

**DOI:** 10.1371/journal.pone.0007176

**Published:** 2009-09-24

**Authors:** Haley D. M. Wyatt, Allison R. Tsang, Deirdre A. Lobb, Tara L. Beattie

**Affiliations:** Southern Alberta Cancer Research Institute and Department of Biochemistry and Molecular Biology, University of Calgary, Calgary, Alberta, Canada; Duke University, United States of America

## Abstract

**Background:**

Telomerase is a reverse transcriptase that maintains the telomeres of linear chromosomes and preserves genomic integrity. The core components are a catalytic protein subunit, the telomerase reverse transcriptase (TERT), and an RNA subunit, the telomerase RNA (TR). Telomerase is unique in its ability to catalyze processive DNA synthesis, which is facilitated by telomere-specific DNA-binding domains in TERT called anchor sites. A conserved glutamine residue in the TERT N-terminus is important for anchor site interactions in lower eukaryotes. The significance of this residue in higher eukaryotes, however, has not been investigated.

**Methodology/Principal Findings:**

To understand the significance of this residue in higher eukaryotes, we performed site-directed mutagenesis on human TERT (hTERT) Q169 to create neutral (Q169A), conservative (Q169N), and non-conservative (Q169D) mutant proteins. We show that these mutations severely compromise telomerase activity *in vitro* and *in vivo*. The functional defects are not due to abrogated interactions with hTR or telomeric ssDNA. However, substitution of hTERT Q169 dramatically impaired the ability of telomerase to incorporate nucleotides at the second position of the template. Furthermore, Q169 mutagenesis altered the relative strength of hTERT-telomeric ssDNA interactions, which identifies Q169 as a novel residue in hTERT required for optimal primer binding. Proteolysis experiments indicate that Q169 substitution alters the protease-sensitivity of the hTERT N-terminus, indicating that a conformational change in this region of hTERT is likely critical for catalytic function.

**Conclusions/Significance:**

We provide the first detailed evidence regarding the biochemical and cellular roles of an evolutionarily-conserved Gln residue in higher eukaryotes. Collectively, our results indicate that Q169 is needed to maintain the hTERT N-terminus in a conformation that is necessary for optimal enzyme-primer interactions and nucleotide incorporation. We show that Q169 is critical for the structure and function of human telomerase, thereby identifying a novel residue in hTERT that may be amenable to therapeutic intervention.

## Introduction

**Figure 1:**
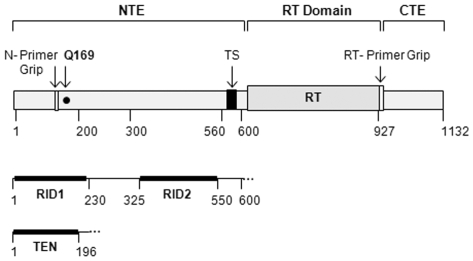
Linear representation of hTERT. The conserved catalytic reverse transcriptase (RT) domain is depicted with a light grey box, the black box represents the conserved telomerase-specific (TS) motif, white boxes depict the N- and RT-primer grip regions and the black circle signifies the approximate location of Q169. The schematics below indicate the predicted locations of RNA-interacting domains 1 and 2 (RID 1 and RID 2, respectively) and the TERT essential N-terminal (TEN) domain. NTE: N-terminal extension and CTE: C-terminal extension.

Telomeres are DNA-protein structures that define the ends of linear chromosomes and form a protective ‘cap’ that enables cells to maintain chromosome length and stability by preventing telomere degradation, recombination, and end-to-end fusions (reviewed in [1], [2]). The proper maintenance of telomere structure and function is crucial for prolonged cell proliferation and genome stability [3]. Indeed, one hallmark of human cancer cells is the up-regulation of telomere maintenance mechanisms that prevent telomere shortening and confer unlimited replicative capacity. In normal cells, however, the inability of conventional DNA polymerases to fully replicate the ends of linear chromosomes results in telomere erosion during cell division [4], [5], [6], [7]. Most eukaryotes utilize an enzyme called telomerase to synthesize and maintain telomeric DNA. Telomerase is a ribonucleoprotein (RNP) reverse transcriptase (RT) complex that minimally contains a catalytic protein subunit, the telomerase reverse transcriptase (TERT), and an RNA subunit, the telomerase RNA (TR) [8], [9], [10]. TERT uses a small RNA template within TR to reverse transcribe telomeric nucleotides onto the ss 3′-ends of chromosomes *in vivo* or G-rich oligonucleotides *in vitro* (reviewed in [11], [12]). TERT proteins are structurally defined by conserved RT and telomerase-specific domains [11]. As illustrated in Figure 1, the structural organization of TERT can be divided into at least three modular regions: 1) a long N-terminal extension (NTE) that contains conserved domains and an unstructured linker region, 2) a central catalytic RT domain with seven evolutionarily-conserved RT motifs, and 3) a short C-terminal extension (CTE) [11], [13]. TERT-specific domains in the NTE and CTE contribute to the biochemical properties that distinguish telomerase from prototypical RT's [11].

Genetic and somatic mutations that disrupt the normal assembly and function of the human telomerase RNP are associated with a spectrum of disease states [14]. The physiological importance of telomerase is further illustrated by the fact that at least 85% of human cancers exhibit up-regulated telomerase activity, which prevents telomere shortening and confers these cells with unlimited replicative capacity. This emphasizes the need for therapeutics that can modulate telomerase activity *in vivo*. Unfortunately, the applicability of current therapies is limited because the molecular mechanism(s) that regulate the assembly and activity of human telomerase are poorly understood. Thus, structure-function studies of human telomerase are necessary for the rationale design of novel telomerase inhibitors.

Functional human telomerase can be reconstituted in rabbit reticulocyte lysates (RRL) by co-expressing human TERT (hTERT) and TR (hTR) [15], [16], although additional proteins are required for telomerase assembly, activity, and regulation *in vivo* [17], [18], [19], [20], [21]. A unique attribute of telomerase is its ability to processively synthesize telomeric repeats (repeat addition processivity) [8], [22], [23]. Telomerase recognizes, binds, and correctly orientates the telomeric DNA primer in the active site to initiate reverse transcription. TERT subsequently copies the TR template, one nucleotide (nt) at a time, until reaching the template 5′-boundary region. At this point, the position of the RT active site relative to the nascent DNA 3′-end must be reset to initiate the next round of telomere synthesis [11]. An important requisite of processive repeat synthesis is that telomerase remains bound to the telomere during the entire reaction cycle. TERT contains template-independent DNA-binding regions outside the RT domain, known as anchor sites, that interact with primer nt upstream of the RNA-DNA hybrid [24], [27]. Anchor site interactions are thought to confer repeat addition processivity by anchoring telomerase to the telomere and preventing dissociation during translocation when the RNA-DNA hybrid in the active site is separated [11].

A telomerase anchor region has been physically and functionally mapped to the TERT N-terminus in ciliates, yeast, and humans [11], [28], [29], [30], [31], [32]. The structure of an N-terminal domain of *Tetrahymena* TERT, known as the conserved TERT essential N-terminal (TEN) domain, has been determined by X-ray crystallography [33]. Conserved residues on the surface of this domain are predicted to form a previously unrecognized ssDNA-binding channel [33]. Photo-cross-linking studies have shown that the *Tetrahymena* TERT (tTERT) TEN domain binds telomeric ssDNA [30], [33]. Mutagenesis of key residues thought to be involved in ssDNA-binding, such as an invariant Gln (Q168 in tTERT), significantly reduced the interaction between tTERT and telomeric ssDNA and impaired enzyme activity *in vitro* [30], [33]. Furthermore, mutation of the corresponding residue in *Saccharomyces cerevisiae* TERT (Est2p Q146A) severely impaired telomerase activity and caused growth defects and telomere loss *in vivo* [31]. It is thought that this Gln is important for telomeric primer recognition and orientation in the catalytic site to initiate telomere synthesis [30], [33]. The contribution of this residue to telomerase structure and function in higher eukaryotes, however, has not been investigated.

In this study, we aimed to determine if the evolutionarily-conserved Gln was important for telomerase function in higher eukaryotes and therefore, characterized the corresponding residue in human TERT (Q169). Site-directed mutagenesis was used to create neutral (Q169A), conservative (Q169N), and non-conservative (Q169D) mutant hTERT proteins. Human telomerase reconstituted with hTERT Q169A, Q169D, or Q169N exhibited severely reduced levels of DNA synthesis *in vitro* and hTERT Q169A failed to catalyze telomere elongation *in vivo*. Substitution of hTERT Q169 dramatically impaired the ability of human telomerase to incorporate nucleotides at the second position of the template. Mutation of Q169 did not abrogate the ability of hTERT to interact with hTR or telomeric ssDNA. When compared to wild type hTERT, however, the Q169 mutants exhibited subtle differences in the relative strength of the interaction with telomeric ssDNA, thereby elucidating a role for Q169 in protein-DNA interactions. Limited proteolysis experiments indicate that the Q169 substitutions altered the protease-sensitivity of the hTERT N-terminus, revealing the importance of conformational changes in this region of hTERT for enzyme activity. We show that the conserved Gln is a functionally critical residue in human telomerase and provide the first detailed evidence regarding the biochemical and cellular significance of this residue in higher eukaryotes.

## Materials and Methods

### Oligonucleotides

All oligonucleotides were synthesized by University Core DNA Services (University of Calgary, Calgary, AB). The oligonucleotide sequences, written 5′ to 3′, are listed in Table 1. Unless otherwise noted, the oligonucleotides used in the primer binding and conventional telomerase activity assays contain biotin at the 5′-end. The oligonucleotides used in the telomere repeat amplification protocol and terminal restriction fragment analysis do not contain biotin tags.

**Table 1:** Description of the ssDNA primers used in the primer binding and conventional telomerase activity assays.

Primer	Length	Biotin	Sequence
bio-TELO30	30	Yes	TTAGGG TTAGGG TTAGGG TTAGGG TTAGGG
bio-TELO24	24	Yes	TTAGGG TTAGGG TTAGGG TTAGGG
bio-TELO18	18	Yes	TTAGGG TTAGGG TTAGGG
bio-TELO12	12	Yes	TTAGGG TTAGGG
bio-TELO6	6	Yes	TTAGGG
bio-antiTELO18	18	Yes	AATCCC AATCCC AATCCC
bio-pBR	24	Yes	AGCCAC TATCGA CTACGC GATCAT
bio-BBP	24	Yes	AATCCG TCGAGC AGAAAT CCGCAA
bio-*Tetrahymena*	18	Yes	TTGGGG TTGGGG TTGGGG
bio-Yeast	15	Yes	TGTGTG GTGTGT GGG
TELO24	24	No	TTAGGG TTAGGG TTAGGG TTAGGG
TELO18	18	No	TTAGGG TTAGGG TTAGGG

Primer, oligonucleotide name; Length, number of nucleotides; Biotin, whether the primer is 5′-biotinylated; Sequence, the 5′ to 3′ nucleotide sequence.

### hTERT constructs

hTERT constructs were cloned using site-directed mutagenesis. Each construct contained an N-terminal *Eco*RI restriction site and FLAG epitope, and a C-terminal SalI restriction site. The identity of each hTERT construct was confirmed by *Eco*RI-*Sal*I digestion, DNA sequencing, and *in vitro* expression of [^35^S]cysteine-labelled hTERT.

### Synthesis and purification of hTR

The full length human telomerase RNA spanning nt 1 to 451, was cloned as described previously [34], and synthesized *in vitro* using the MEGAscript T7 transcription kit (Ambion, Cedarlane® Laboratories Ltd.) as detailed in [28].

### *In vitro* reconstitution of human telomerase

hTR was cloned and synthesized as described previously [28], [34]. Human telomerase was reconstituted *in vitro* with the rabbit reticulocyte lysate (RRL) TNT^®^ T7 Transcription-Translation System (Promega, Fisher Scientific Ltd.), according to the manufacturer's instructions. Final reaction volumes containing the appropriate hTERT plasmid DNA (10 ng/µL) and full length *in vitro*-transcribed hTR (10 ng/µL) were incubated at 30°C for 90 or 120 min.

### Cell lines and culture conditions

Simian immunodeficiency virus 40 (SV40) large T-antigen transformed human embryonic kidney cells (293T) were cultured in GIBCO^®^ high glucose Dulbecco's Modified Eagle's Media (DMEM) supplemented with 10% fetal bovine serum, 50 U/mL penicillin G, and 50 µg/mL streptomycin sulfate (Invitrogen). 1 µg/mL puromycin (Sigma-Aldrich) was added to the growth media for stable 293T cell lines. 293T cells were continuously passaged at 1∶5 or 1∶6.

Primary human foreskin fibroblasts (BJ cells) were cultured in Lonza BioWhittaker* Minimum Essential Medium Eagle (EMEM) with EBSS (Fischer Scientific Ltd.) supplemented with L-glutamine, 10% fetal bovine serum, 50 U/mL penicillin G, and 50 µg/mL streptomycin sulfate (Invitrogen). Stable cell lines were cultured in media containing 0.1–0.5 µg/mL puromycin (Sigma-Aldrich). BJ cells were passaged at 1∶4 or 1∶5 until the culture divided 2.5 times more than the vector control cells or until the culture had exhausted its replicative potential. This stage was defined as the period when cultures failed to become confluent within 30 days and stained positive for β-galactosidase at pH 6.0.

### Cell transfections and retroviral infections

293T cells were stably transfected with 1 µg of pBABE_puro_, pBABE_puro_-FLAG-hTERT WT, or pBABE_puro_-FLAG-hTERT Q169A DNA using FuGENE transfection reagent (Roche). Transfected cultures were split into selective media containing 1.0 µg/mL puromycin (Sigma-Aldrich) 48 h post-transfection. The first plate to reach confluence under selection was arbitrarily defined as mean population doubling (mpd) 0.

Retroviral infections were used to establish polyclonal BJ cell lines that stably expressed pBABE_puro_, pBABE_puro_-FLAG-hTERT WT, or pBABE_puro_-FLAG-hTERT Q169A. 293T cells were used to package amphotropic retroviruses created with the helper plasmid pCL-10A1 and pBABE_puro_, pBABE_puro_-FLAG-hTERT WT, or pBABE_puro_-FLAG-hTERT Q169A plasmid DNA. 293T cells were transiently transfected with the appropriate construct DNA using FuGENE transfection reagent (Roche). The viral supernatant was collected 24 h after transfection, filtered, supplemented with polybrene (Sigma-Aldrich) to a final concentration of 8 µg/mL, and applied to BJ cells. 48 h after retroviral infection, BJ cells were split 1∶4 with selective media containing 0.1–0.5 µg/mL puromycin (Sigma-Aldrich). Mean population doubling 0 was arbitrarily defined as the time point when the infected cells reached 80–90% confluency in selective growth media.

### Cell lysis

Cell pellets were lysed in NP-40 lysis buffer (10 mM Tris-HCl, pH 7.5; 1% [vol/vol] NP-40; 10% [vol/vol] glycerol; 1 mM EGTA; 1 mM MgCl_2_; 150 mM NaCl) supplemented with fresh 5 mM β-mercaptoethanol, 10 U/mL RNase Out (Invitrogen), and complete EDTA-free proteinase inhibitor cocktail tablet (Roche) on ice for 30 min followed by rocking at 4°C for an additional 30 min. Cell lysates were centrifuged at 12 100 × g for 30 min at 4°C to remove the insoluble debris. The soluble fraction was incubated with pre-equilibrated Protein G Sepharose™ (GE Healthcare) for 30 min at 4°C with rocking and then centrifuged at 604 × g for 3 min at 4°C. Protein concentration in the pre-cleared soluble fraction was determined using the Bio-Rad Protein Detection Assay (Bio-Rad).

### Immunoprecipitations

FLAG-hTERT was immunoprecipitated from soluble cell lysate (800–1000 µg) with anti-FLAG M2 affinity resin (Sigma-Aldrich) in IP buffer 150 (10 mM Tris-HCl, pH 7.5; 1% [vol/vol] NP-40; 10% [vol/vol] glycerol; 1 mM EGTA; 1 mM MgCl_2_; 150 mM NaCl) containing 10 U/mL RNase Out (Invitrogen) and EDTA-free proteinase inhibitor cocktail (Roche) for 2 h at 4°C with rocking. The protein-bead complexes were washed four times with either 1 mL of IP buffer 150 (washes 1 and 4) or IP buffer 300 (IP buffer containing 300 mM NaCl; washes 2 and 3). FLAG-hTERT was eluted from the affinity resin by two rounds of competitive elution with excess 3× FLAG peptide (Sigma-Aldrich); beads were resuspended in 30 µL IP 150 buffer containing 2 mg/mL 3× FLAG peptide, rocked for 30 min at 4°C, and then centrifuged at 604 × g for 3 min (4°C).

### Western analysis

hTERT protein expression was analyzed in immunoprecipitates from 293T and BJ cells. SDS-PAGE sample buffer was mixed with 30 µL of 3 × FLAG peptide eluate and the samples were boiled for 5 min and resolved by 8% SDS-PAGE. The gel was transferred to a polyvinylidene diflouride membrane in transfer buffer (25 mM Tris base; 190 mM glycine; 20% [vol/vol] methanol; pH adjusted to 8.0), fixed in methanol, air dried, and blocked with TBS (50 mM Tris-HCl, pH 7.4; 150 mM NaCl) containing 0.5% Tween-20 and 5% (wt/vol) nonfat dry milk (NFDM) for 1–2 h at RT. To detect hTERT, blocked membranes were probed with a 1∶1000 dilution of the polyclonal anti-hTERT antibody [20] (a generous gift from Dr. S. Artandi, Stanford School of Medicine, Stanford, CA) prepared in TBST (TBS; 0.1% Tween-20) containing 2.5% (wt/vol) NFDM overnight at 4°C with gentle rocking. Blots were washed three times for 15–20 min each in TBST at RT, rinsed in TBS, and incubated with ECL™ anti-rabbit immunoglobin G, horseradish peroxidase linked (from donkey) secondary antibody (GE Healthcare) (1∶2000 dilution in TBST-2.5% [wt/vol] NFDM) for 1 h at RT. The blots were subsequently washed three times (10–15 min at RT) in TBST and once in TBS (5–10 min). Proteins were detected with enhanced chemiluminescence (ECL) reagent (GE Healthcare), according to the manufacturer's instructions.

### hTERT-hTR coimmunoprecipitation

hTR was transcribed from an *Eco*RI-linearized plasmid in the presence of [α-^32^P]UTP (3000 Ci/mmol; 10 mCi/mL; GE Healthcare) with the MEGAscript T7-coupled expression system (Ambion, Cedarlane^®^ Laboratories Ltd.), according to the manufacturer's instructions. Unincorporated nucleotides were removed using G-25 gel filtration microspin columns (GE Healthcare). Human telomerase was reconstituted using the RRL TNT^®^ T7 Transcription-Translation System (Promega, Fisher Scientific Ltd.) with 30 ng/uL [^32^P]UTP-labelled hTR, 60 ng/uL FLAG-hTERT WT, Q169A, Q169D, or Q169N plasmid DNA, and [^35^S]cysteine (>1000 Ci/mmol, 10 mCi/mL; GE Healthcare). RRL reactions were incubated at 30°C for 60–90 min. hTERT-hTR complexes were immunoprecipitated using a method similar to that described previously [35].

### Limited proteolysis

α-chymotrypsin from bovine pancreas (Sigma-Aldrich) was reconstituted in 1 mM HCl containing 2 mM CaCl and diluted to the appropriate working concentration in 1× reaction buffer (10× stock  = 200 mM Tris-HCl, pH 8.3; 150 mM MgCl_2_; 630 mM KCl; 0.5% Tween-20; 10 mM EGTA, pH 8.0). RRL containing [^35^S]cysteine-labelled full length or 1–300 FLAG-hTERT WT, Q169A, Q169D, or Q169N was digested for 2 min at 30°C in 1× reaction buffer containing 10, 100, or 1000 ng/mL chymotrypsin (Sigma-Aldrich). 15 µL proteolysis reactions contained comparable amounts of [^35^S]cysteine-labelled hTERT. Reactions were terminated by the addition of SDS-PAGE loading buffer and boiling (5 min). Following digestion, products were resolved by 12% SDS-PAGE and visualized by autoradiography and phosphorimaging.

### Telomere repeat amplification protocol

Telomerase activity was detected using a modified, two-step version of the telomeric repeat amplification protocol (TRAP) [36]. In the first step (telomere extension), 1 µL of RRL or 2 µL of 3 × FLAG peptide eluate was incubated for 30 min at RT in a 50 µL volume containing 1× reaction buffer (10× stock  = 200 mM Tris-HCl, pH 8.3; 150 mM MgCl_2_; 630 mM KCl; 0.5% Tween-20; 10 mM EGTA, pH 8.0), 2.5 mM dATP, dCTP, dGTP, and dTTP, 0.1 µg TS primer (5′-AATCCGTCGAGCAGAGTT-3′), and 2 U of *Taq* DNA polymerase (Invitrogen). In the second step (amplification), 0.1 µg of ACX primer (5′-GCGCGG[CTTACC]_3_CTAACC-3′) and 1 µL of [α-^32^P]dGTP (3000 Ci/mmol; 10 mCi/mL; GE Healthcare) was added to each 50 µL extension reaction, which was subsequently PCR-amplified for 20–25 cycles consisting of 95°C for 30 s, 50°C for 30 s, and 72°C for 90 s. TRAP reaction products (9 µL) were electrophoresed on a nondenaturing 12% (wt/vol) polyacrylamide gel (29∶1 [wt/wt] acrylamide-bisacrylamide) and detected by autoradiography or exposure to a phosphorimaging screen.

### Conventional telomerase activity assay

The conventional telomerase activity (CTA) assay was used to measure the activity of human telomerase reconstituted in RRL, as described previously [28]. The processivity of human telomerase was determined with pulse-chase conventional telomerase activity (CTA) assays. In these experiments, RRL containing reconstituted telomerase was incubated in a 40 µL reaction containing 1× CTA buffer (10 × stock  = 500 mM Tris–HCl, pH 8.3; 500 mM KOAc; 10 mM MgCl_2_; 50 mM β-mercaptoethanol; 10 mM spermidine), 1.5 µM biotinylated (TTAGGG)_3_ primer, 1 mM dATP, 1 mM dTTP, 1.25 µM dGTP, and 6 µL [α-^32^P]dGTP (3000 Ci/mmol, 10 mCi/mL; GE Healthcare) at 30°C for 5 min (pulse). The pulse reaction was chased with 200 µM non-biotinylated (TTAGGG)_3_ primer for 5, 15, or 30 min (chase) and terminated by incubating with stop buffer (10 mM EDTA; 2 M NaCl; 0.1 mg/mL RNase A) at 37°C for 15 min. Elongated biotinylated pulse primers were separated from elongated non-biotinylated chase primers using the method described previously [28].

Telomerase activity was measured using data collected with the standard CTA assay and processivity was calculated with the data obtained in pulse-chase CTA experiments. Using phosphoimager analysis and Quantity One^®^ version 4.5.0 software (Bio-Rad), the signal of each elongation product was quantified and normalized to the total number of labeled dGTP incorporated. Telomerase activity within one hexameric repeat (P_i_) was calculated using the formula P_i_  =  (T_i_ + T_i+1_ + … + T_i+5_). T values correspond to the signal of the elongation product at position i. The normalized values obtained at positions T_i_ … T_i+5_ in each independent experiment were added and expressed as a fraction of WT telomerase activity. The mean activity was determined from three independent experiments and the results are reported±SEM. GraphPad InStat version 3.00^®^ (GraphPad Software) was used to calculate Student's two-tailed unpaired T-tests and determine if total DNA synthesis was statistically different for human telomerase reconstituted with hTERT WT versus Q169A, Q169D, or Q169N.

Repeat addition processivity (P_i_) was calculated using methods similar to those described previously [37], [38]. The normalized signals of the +6, +12, +18, +24, +30, and +36 elongation products were applied to the formula P_i_  =  (T_i+6_)/(T_i_ + T_i+6_). First, repeat addition processivity was calculated for each pair of elongation products. Next, the repeat addition processivity values at each position were summed to determine the overall processivity. The overall processivity results of three independent experiments were averaged and the SEM was calculated. Average repeat addition processivity for mutant telomerases was then expressed relative to the wild type enzyme and the SEM was calculated. Statistical significance was assessed using GraphPad InStat version 3.00^®^ (GraphPad Software).

### Primer binding assay

hTERT was synthesized in the absence of hTR with the RRL TNT^®^ T7 Transcription-Translation System (Promega, Fisher Scientific Ltd.), as described in [28]. [^35^S]cysteine-labelled hTERT proteins were tested for the ability to interact with 5′-biotinylated oligonucleotides using a slightly modified version of the previously described DNA-binding assay [28], in which the primer mix was incubated on ice for 45–60 min prior to adding [^35^S]cysteine-labelled hTERT. Primer binding results were analyzed as described [28], with the exception that reaction products were quantified by phosphoimager analysis.

### Telomere restriction fragment analysis

Telomeres were visualized by in-gel hybridization of *Hin*fI and *Rsa*I restriction enzyme-digested genomic DNA (5 µg) with a telomeric DNA probe (generously provided by Dr. J. Karlseder, Salk Institute for Biological Studies, La Jolla, CA), (TTAGGG)*_x_*, labelled with Klenow and [α-^32^P]dGTP (3000 Ci/mmol, 10 mCi/mL; GE Healthcare). Aliquots of digested DNA were electrophoresed through a 0.6% agarose gel at 500 Vh. Gels were denatured for 45 min in denaturation solution (0.5 M NaOH; 1.5 M NaCl), neutralized for 45 min in neutralization solution (1 M Tris, pH 8.0; 1.5 M NaCl), and dried at RT (45 min) and 60°C (45 min). The dried gel was subsequently denatured for 10 min and neutralized for 10 min before hybridization in 5× SSC (20× stock  = 3 M NaCl; 300 mM sodium citrate) containing^ 32^P-labelled telomeric probe at 37°C for 20 h. Hybridized gels were washed three times in 2× SSC for 10 min (RT), washed once in nuclease-free water for 5 min (RT), dried for 20 min at 60°C, and exposed to a phosphoimager screen overnight. Mean telomere length was calculated from at least three independent experiments using OptiQuant™ version 5.0 software (PerkinElmer, Inc.).

### β-galactosidase staining

Adherent BJ cells grown in 10 cm culture dishes were washed in PBS (pH 7.2), fixed for 3–5 min at RT in PBS (pH 7.2) containing 0.5% gluteraldehyde (Sigma-Aldrich), washed with PBS (pH 7.2) supplemented with 1 mM MgCl_2_, and stained overnight at 37°C (no CO_2_) with fresh β-galactosidase staining solution: PBS (pH 6.0) containing 1 mg/mL 5-bromo-4-chloro-3-indolyl β-D-galactoside (X-Gal) (stock  = 20 mg/mL in dimethylformamide), 1 mM MgCl_2_, 120 µM potassium ferricyanide, and 120 µM potassium ferrocyanide. Staining was evident the next day; stained cells were washed in PBS (pH 7.2) and visualized with bright-field microscopy.

## Results

### Q169 mediates the polymerase function of human telomerase *in vitro*

**Figure 2:**
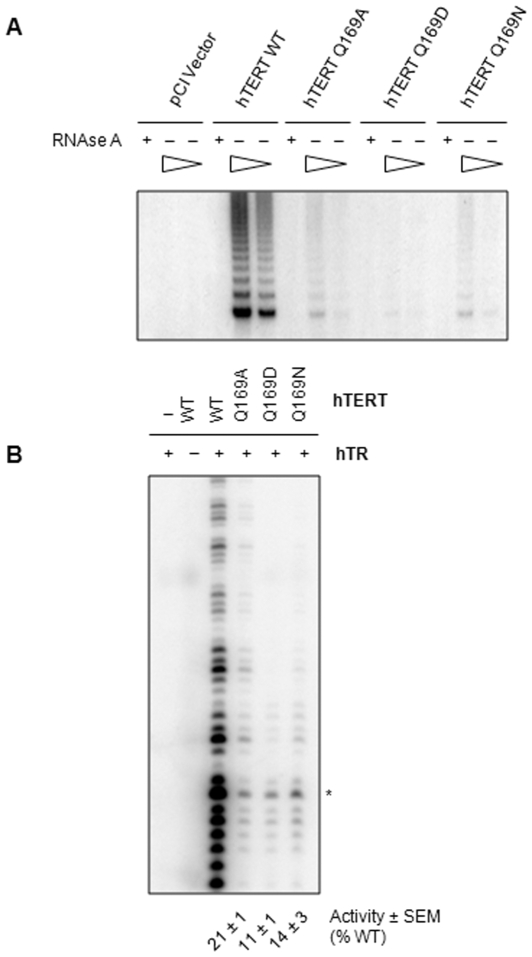
*In vitro* telomerase activity of hTERT Q169 mutants. *A,* Human telomerase was reconstituted in rabbit reticulocyte lysates (RRL) with hTR and FLAG-hTERT WT, Q169A, Q169D, or Q169N and tested for *in vitro* enzyme activity using the telomere repeat amplification protocol. RRL reconstitution reactions (1 µL) were assayed in the presence and absence of 5 µg RNAse A. The triangles represent a five-fold dilution of each reaction to confirm that the amplification assays were in the linear range. *B,* Human telomerase reconstituted in RRL with hTR and FLAG-hTERT WT, Q169A, Q169D, or Q169N was tested for activity using the conventional telomerase activity assay and an 18 nt canonical human telomeric primer, bio-TELO18. Mean telomerase activity within the first hexameric repeat was calculated from three independent experiments and reported relative to the WT enzyme. Mean values±SEM are shown at bottom of each lane. The asterisk indicates 22 nt elongation products, as determined by comparison with the mobility of a 5′-end-radiolabelled 18 nt telomeric primer containing 3′-biotin (not shown).

We first determined whether the evolutionarily-conserved Gln residue in the TEN domain was required for human telomerase activity *in vitro* using the telomere repeat amplification protocol (TRAP). We found that human telomerase reconstituted with hTERT Q169A, Q169D, or Q169N exhibited a dramatic reduction in catalytic activity compared to the WT enzyme (Figure 2A). Control reactions showed that RNase A inhibited these reactions, indicating that DNA synthesis was catalyzed by telomerase and not by contaminating polymerases present in RRL. The Q169D substitution was particularly disruptive and largely eliminated enzymatic activity, perhaps due to electrostatic repulsion between the polyanionic DNA backbone and electronegative carboxylic acid side-chain.

The TRAP is a highly sensitive method to detect telomerase activity. However, it does not distinguish between enzymes with catalytic defects (*i.e.* impaired DNA polymerization) and non-processive enzymes with translocation defects that synthesize less than four telomeric repeats [36], [39]. Therefore, we used the conventional telomerase activity assay (CTA) to gain more insight into the catalytic defects of the hTERT Q169 mutants. Similar to the TRAP results, human telomerase reconstituted with hTERT Q169A, Q169D, or Q169N exhibited markedly reduced activity compared to the WT enzyme when tested with an 18 nt telomeric ssDNA primer, bio-TELO18 (Figure 2B and Table 1). CTA assays were performed with bio-TELO18 because human telomerase exhibits robust activity with this primer [28], although we observed similar catalytic defects with a shorter primer, bio-TELO6 (data not shown). Quantification of three independent experiments showed that telomerase reconstituted with hTERT Q169A was approximately 20% as active as the WT enzyme (p<0.001) (Figure 2B). The activity defect was more severe for telomerases reconstituted with either hTERT Q169D or hTERT Q169N, which were only 11% and 14% as active as WT telomerase, respectively (p<0.001). We observed a striking defect in the ability of the Q169 mutants to incorporate the second nucleotide, which corresponds to the second G in the extension product 5′-GGGTTAG-3′. This could be explained if the Q169 mutants have reduced affinity for the nucleotide that would be incorporated at this position. However, we have been unable to address this question because kinetic studies and derivation of affinity constants cannot be performed with RRL-reconstituted human telomerase. This is due to the high abundance of endogenous RRL proteins and the inability to accurately quantify the concentration of functionally-competent telomerase. Similar arguments exist for FLAG-immunoprecipitated human telomerase. An alternative, but not necessarily independent explanation is that substitution of Q169 altered hTERT conformation, which prevented efficient nucleotide incorporation (below).

**Figure 3:**
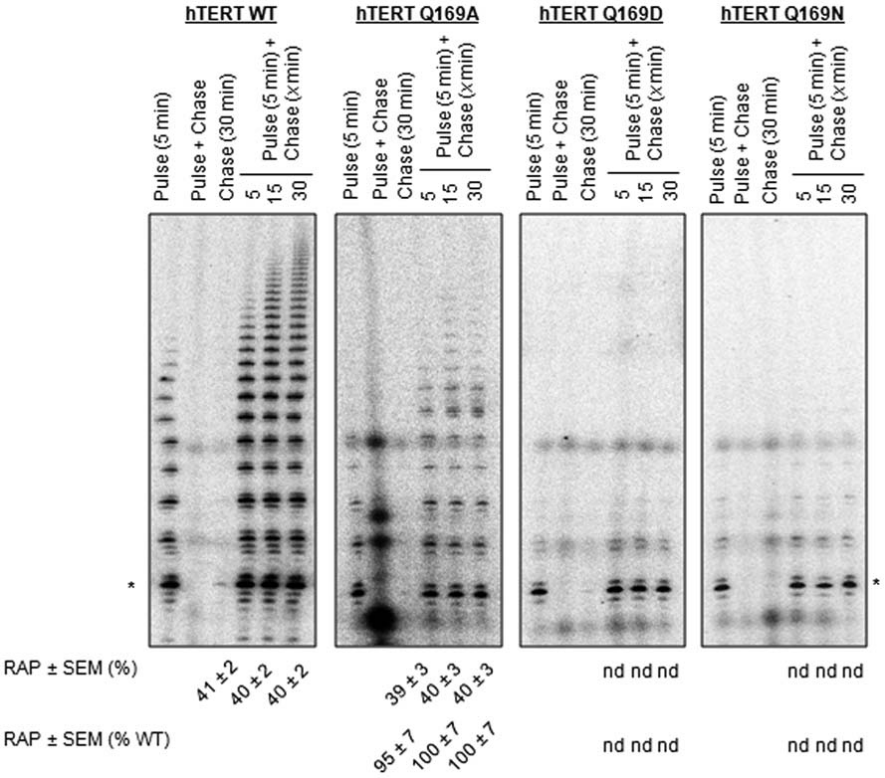
Repeat addition processivity of hTERT Q169 mutants. Pulse-chase conventional telomerase activity assays were used to measure the repeat addition processivity of telomerase with bio-TELO18. The telomerase reaction was initiated with 1.5 µM bio-TELO18. Telomerase was allowed to extend bio-TELO18 for 5 min and then challenged with 200 µM chase primer (TELO18) for 5, 15, and 30 min. The first three lanes in each panel represent control reactions (left to right): pulse (5 min), reactions performed with pulse primers alone to show that telomerase can efficiently elongate this primer; pulse + chase, pulse and chase primers added simultaneously to demonstrate the efficiency of the chase conditions; chase (30 min), reactions performed with the chase primer alone to show that the non-biotinylated reaction products were not isolated on streptavidin beads. Repeat addition processivity within the first five repeats was calculated from three independent experiments. Mean values are reported±SEM at the bottom of each lane. Asterisks denote the position of the 22 nt elongation product, as determined by comparison with the mobility of a 5′-end-radiolabelled 18 nt telomeric oligonucleotide harboring a 3′-biotin residue (not shown). *nd*, not determined.

Pulse-chase CTA experiments indicated that mutation of hTERT Q169 did not affect repeat addition processivity (Figure 3). A direct correlation was observed between the length of the extended pulse primer and the time that WT and Q169A telomerase was incubated with chase DNA, indicating that telomerase remained associated with bio-TELO18 and catalyzed processive DNA synthesis. Quantification of the repeat addition processivity within the first five telomeric repeats revealed that hTERT Q169A was at least 95% as processive as WT telomerase when pulsed with bio-TELO18 and chased with TELO18 (p>0.05). This data suggests that hTERT Q169 has a critical role in the first round of DNA synthesis but is less important for subsequent rounds of DNA synthesis (*i.e.* translocation). However, we cannot rule out a potential role for Q169 in repeat addition processivity since were unable to quantify the processivity of telomerase reconstituted with hTERT Q169D or Q169N.

### Biochemical characterization of hTERT Q169

**Figure 4:**
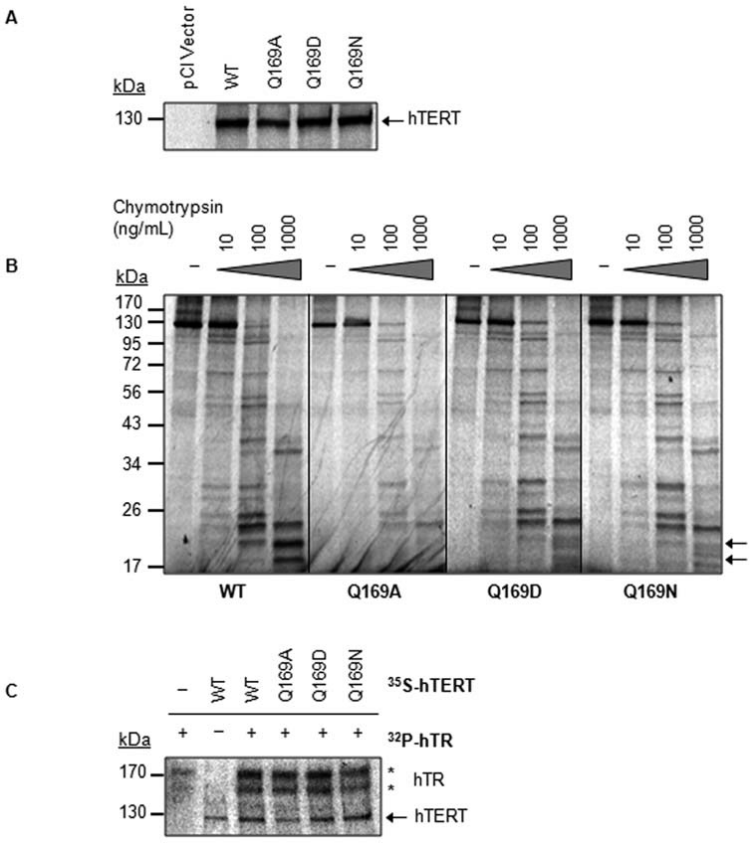
Biochemical analysis of hTERT Q169 mutants. *A,* Mutation of hTERT Q169 does not compromise protein stability. SDS-PAGE and autoradiography was used to assess protein expression and stability in RRL (left to right): pCI vector alone, FLAG-hTERT WT, FLAG-hTERT Q169A, FLAG hTERT Q169D, and FLAG-hTERT Q169N. RRL reactions (2 µL) were resolved by 8% SDS-PAGE and visualized by autoradiography and phosphorimaging. *B,* hTERT Q169 mediates hTERT conformation. Comparable amounts of [^35^S]cysteine-labelled FLAG-hTERT WT, Q169A, Q169D, or Q169N was incubated with 0, 10, 100, or 1000 ng/mL chymotrypsin at 30°C for 2 min and resolved by 12% SDS-PAGE. Arrows indicate the proteolytic fragments that are diminished upon substitution of hTERT Q169. *C,* Substitution of hTERT Q169 does not abrogate hTERT-hTR interactions. [α-^32^P]UTP-labelled hTR (indicated by asterisks) was coimmunoprecipiated with [^35^S]cysteine-labelled FLAG-hTERT WT, Q169A, Q169D, and Q169N (indicated by arrow). Coimmunoprecipitates were resolved by 4–15% SDS-PAGE and visualized by autoradiography and phosphorimaging.

To investigate the basis for the activity defects, we determined whether Q169 modulates hTERT stability, conformation, and physical interactions with hTR. There was no appreciable difference in the protein levels of [^35^S]cysteine-labelled hTERT WT, Q169A, Q169D, or Q169N (Figure 4A), indicating that Q169 mutagenesis does not have a significant impact on hTERT stability *in vitro*. However, it remained possible that Q169 mutation might induce a conformational change in hTERT that inhibited enzyme activity without compromising protein stability. Therefore, RRL containing comparable levels of [^35^S]cysteine-labelled WT or Q169 mutated hTERT was subjected to limited proteolysis using chymotrypsin. The hTERT Q169A, Q169D, and Q169N mutants were found to be more sensitive to chymotrypsin digestion than WT hTERT (Figure 4B). Specifically, the signal intensity of [^35^S]cysteine-labelled proteolytic fragments migrating at approximately 20 kDa was diminished in hTERT Q169A, Q169D, and Q169N. This was an hTR-independent effect because the proteolytic fragments derived from hTERT reconstituted in the presence of hTR were indistinguishable from those observed in its absence (Figure S1). This observation was confirmed with time-course proteolytic digestions of [^35^S]cysteine-labelled hTERT WT, Q169A, Q169D, or Q169N that were synthesized in the presence and absence of hTR (Figure S2).

To gain insight into the identity of the proteolytic fragments at approximately 20 kDa, we performed limited proteolysis on truncated hTERT proteins containing the first 300 amino acids. A comparison of the proteolytic profiles of [^35^S]cysteine-labelled 1–300 hTERT WT and Q169A, Q169D, and Q169N revealed a reduction in the 20 kDa proteolytic fragment in the Q169 mutants, similar to the results obtained with full length hTERT (Figure S3). This provides the first experimental evidence that Q169 is important for the structure of the hTERT N-terminus.

Interactions between hTERT and hTR are essential for telomerase activity and some mutations in hTERT that impair hTR-binding reconstitute nonfunctional enzymes [34], [35], [40]. Thus, defects in hTERT-hTR assembly could potentially explain the loss of activity observed with the hTERT Q169 mutants. To test this possibility, human telomerase was reconstituted with [α-^32^P]UTP-labelled hTR and [^35^S]cysteine-labelled-FLAG-hTERT WT, Q169A, Q169D, or Q169N. [^35^S]cysteine-labelled hTERT was immunoprecipitated with FLAG antibodies and analyzed for [α-^32^P]UTP-labelled hTR. As shown in Figure 4C, the hTERT Q169 mutants bound hTR as efficiently as hTERT WT, indicating that the functional defects observed with these mutants were not due to abrogated hTERT-hTR interactions.

### hTERT Q169 mutants retain sequence-specific ssDNA-binding activity

**Figure 5:**
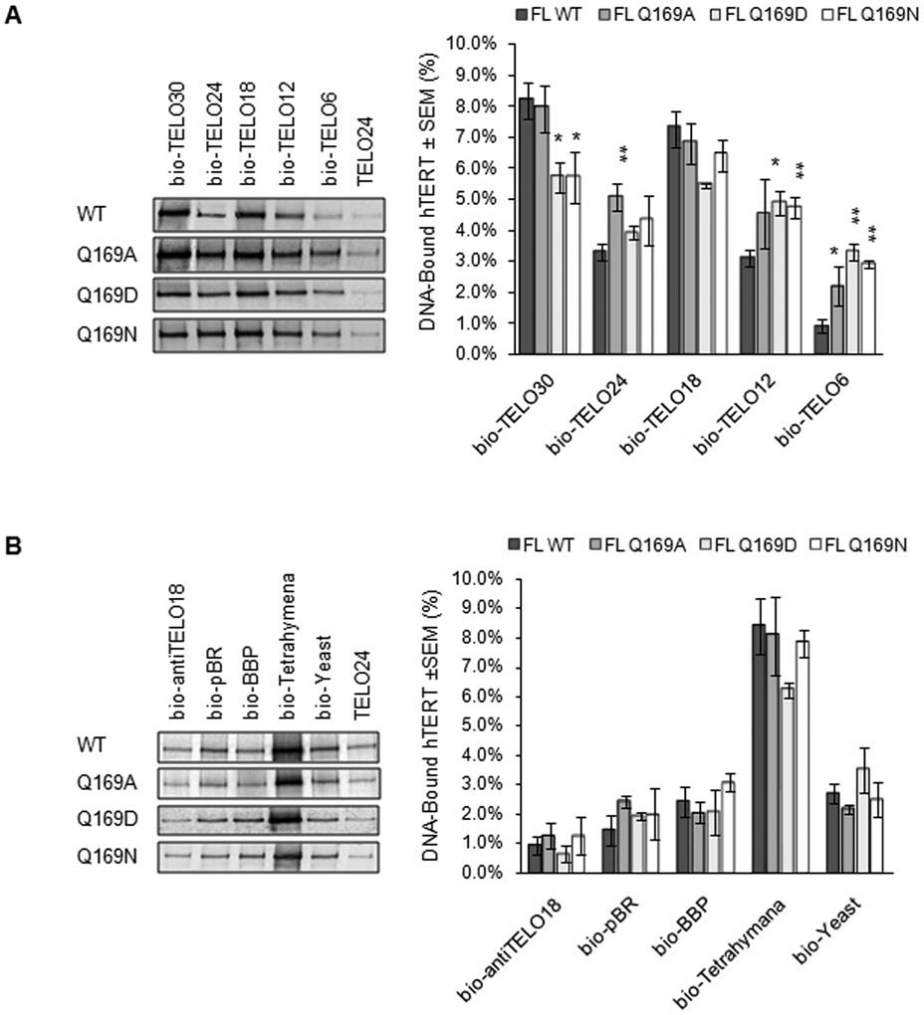
hTERT Q169 mutants interact sequence-specifically with telomeric ssDNA. The primer binding assay was used to investigate interactions between hTERT WT and Q169 mutants and 5′-biotinylated ssDNA primers comprised of *A,* different lengths of human telomeric DNA and *B,* non-human telomeric or random DNA. Primer sequences are listed in Table 1. hTERT-DNA complexes were immobilized on neutravidin beads and separated by 8% SDS-PAGE and visualized with autoradiography and phosphorimaging (left panels). Quantification and statistical analysis of at least three independent experiments is shown in the graphs (right panels). Mean values are reported±SEM. Asterisks denote levels of statistical significance compared to the interaction between hTERT WT and the corresponding primer; p<0.05 (*) and p<0.01 (**).

Telomeric primers have to be positioned correctly within the telomerase active site to initiate reverse transcription. In addition to primer-template interactions, this requires interactions between the TERT RT domain and DNA 3′-end, as well as between the N-terminus (anchor region) and DNA 5′-end [11]. Thus, suboptimal interactions between hTERT and DNA primers could explain the activity defects observed with hTERT Q169 mutants. We used a recently-developed DNA-binding assay to characterize physical interactions between hTERT WT, Q169A, Q169D, and Q169N and 5′-biotinylated ssDNA primers (Table 1) [28]. We found that the Q169 mutants retained sequence-specific DNA-binding activity *in vitro* (Figures 5A and B). When compared with hTERT WT, however, the Q169 mutants exhibited subtle differences in their interactions with specific lengths of human telomeric DNA (Figure 5A). The relative strength of the interaction between short telomeric primers (bio-TELO12 and bio-TELO6) and each of the Q169 mutants was greater than that observed for hTERT WT. This was also observed between hTERT Q169A and bio-TELO24, and may reflect tighter binding of the primers to the Q169 mutants. In contrast, the relative strength of the interaction between hTERT Q169D or Q169N and bio-TELO30, the longest primer we tested, was significantly reduced.

We next tested the Q169 mutants for interactions with non-human telomeric or random ssDNA to determine whether Q169 was important for hTERT's sequence-specificity. As shown in Figure 5B, hTERT Q169A, Q169D, and Q169N demonstrated sequence-specific DNA-binding that was indistinguishable from hTERT WT. The interactions between Q169 mutants and primers containing either 18 nt (bio-antiTELO18) or 24 nt (bio-pBR and bio-BBP) of non-telomeric DNA were not statistically different from those observed with hTERT WT (p>0.05). When tested with primers representing the telomeres of other organisms, hTERT WT and Q169 mutants interacted very efficiently with a primer containing 18 nt of *Tetrahymena* telomeric DNA (bio-Tetrahymena) and much less efficiently with a primer containing 15 nt of *S. cerevisiae* telomeric DNA (bio-Yeast) (Figure 5B). The interaction with bio-Tetrahymena is consistent with the ability of WT human telomerase to elongate primers containing this G-rich sequence (TTGGGG) [22].

### Q169 mediates the ability of truncated hTERT proteins to interact with ssDNA

**Figure 6:**
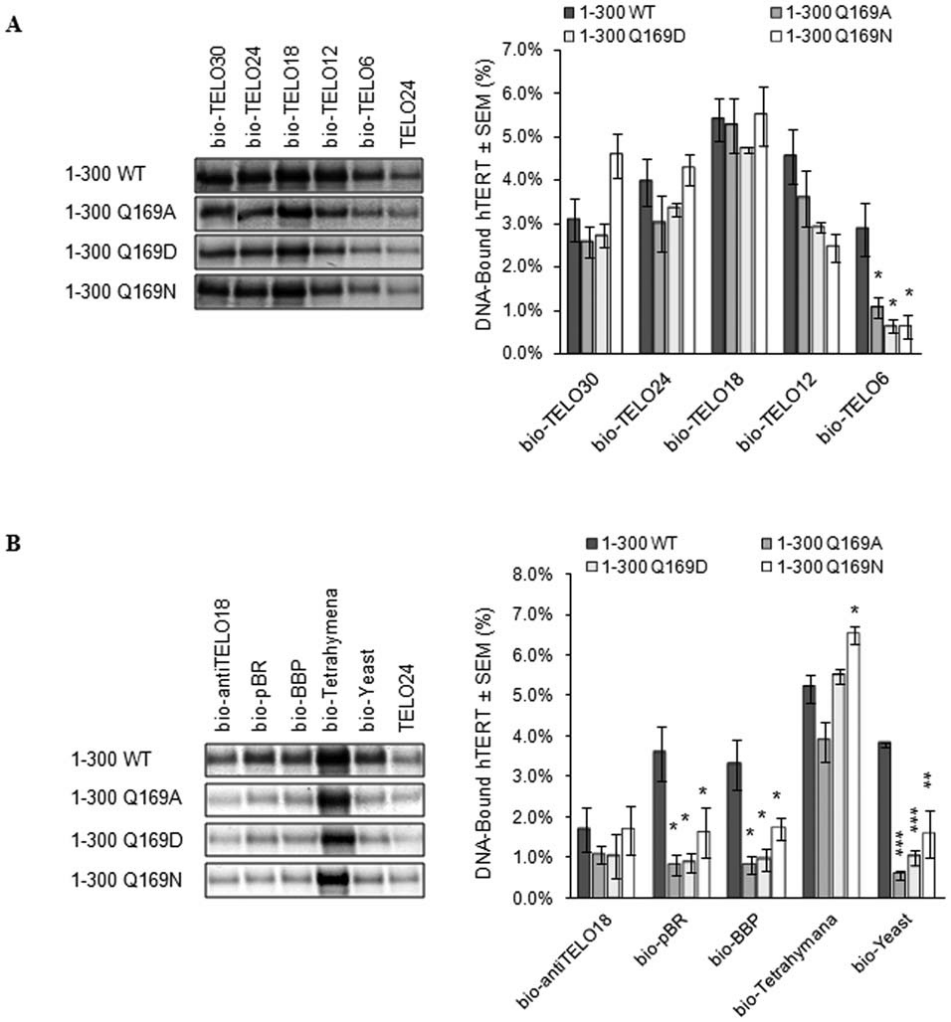
Q169 mediates physical interactions between truncated hTERT proteins and ssDNA. The primer binding assay was used to test the ability of truncated hTERT proteins comprised of amino acids 1–300 (WT and Q169 mutants) to bind 5′-biotinylated ssDNA primers comprised of *A,* different lengths of human telomeric DNA and *B,* non-human telomeric or random DNA (Table 1). hTERT-DNA complexes were immobilized on neutravidin beads and separated by 8% SDS-PAGE and visualized with autoradiography and phosphorimaging (left panels). Quantification and statistical analysis of at least three independent experiments is in graphical format (right panels) and the mean is reported±SEM. Asterisks denote levels of statistical significance compared to hTERT 1–300 WT binding to the corresponding DNA primer; p<0.05 (*), p<0.01 (**), and p<0.001 (***).

TERT contains multiple DNA-binding regions that make it difficult to assess how one amino acid influences DNA-binding [29]. We therefore introduced the Q169 mutations into the isolated hTERT TEN domain (amino acids 1–196) and the tested these mutants for DNA-binding. However, we were unable to detect stable interactions between the TEN domain and telomeric ssDNA primers (data not shown). Therefore, the Q169A, Q169D, and Q169N mutations were cloned into an hTERT fragment comprised of residues 1 to 300 [28]. Each of the hTERT 1–300 constructs exhibited readily apparent ssDNA-binding activity (Figure 6A). However, the Q169 substitutions caused a reduction in the relative strength of the interactions between hTERT 1–300 and short telomeric primers (bio-TELO12 and bio-TELO6). This result provided an interesting contrast to that observed with full length proteins, where mutation of Q169 increased the apparent strength of the interaction with bio-TELO12 and bio-TELO6 (Figure 5A). Furthermore, the 1–300 hTERT Q169 mutants showed a marked decrease in the interaction with primers comprised of yeast telomere (bio-Yeast) or random (bio-pBR and bio-BBP) DNA (Figure 6B). Taken together, the data presented in Figures 6A and B indicate that Q169 is involved in the ssDNA-binding activity of the hTERT N-terminus and thus, contributes to anchor site interactions in human telomerase.

### Q169 mediates the cellular functions of human telomerase

To extend our *in vitro* studies, we determined if Q169 mediates the biological activity of telomerase in human cells. hTERT Q169A was stably expressed in telomerase positive (SV40 large T-antigen transformed embryonic kidney cells; 293T) and telomerase negative (foreskin fibroblasts; BJ) human cells. Q169A was chosen as a representative mutant for these experiments since this substitution was the least detrimental to telomerase activity *in vitro*.

**Figure 7:**
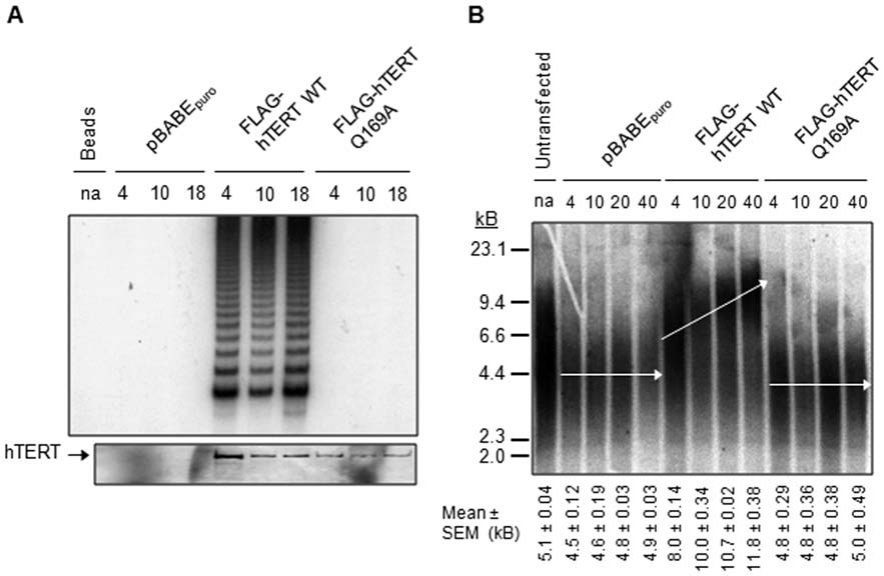
hTERT Q169 is required for telomerase activity and telomere length-maintenance in transformed human cells. *A,* Extracts of 293T cells stably expressing pBABE_puro_, pBABE_puro_-FLAG-hTERT WT, or pBABE_puro_-FLAG-hTERT Q169A were immunoprecipitated with anti-FLAG antibodies. FLAG-hTERT was eluted from the beads by competition with excess 3 × FLAG peptide. 2 µL of eluate was tested for telomerase activity by the telomere repeat amplification protocol (top panel) and 35 µL was resolved by 8% SDS-PAGE and examined for hTERT expression by Western blotting with anti-hTERT antibodies (bottom panel). The mean population doubling is indicated above each lane. *na*, not applicable. *B,* Bulk telomere length was measured using the terminal restriction fragment analysis. Mean telomere length and standard error of the mean (SEM) was calculated from at least three independent experiments and is indicated below each lane. The mean population doubling is indicated above each lane. Arrows are included for visual clarification and represent the approximate mean of each sample.

We first investigated the cellular function of hTERT Q169A in 293T cells. Figure 7A shows that anti-FLAG-immunoprecipitated hTERT WT exhibited robust TRAP activity whereas the Q169A mutant was consistently inactive. The loss of telomerase activity was not due to abrogated protein expression (Figure 7A) or nuclear exclusion (data not shown). As expected, telomere elongation was observed in 293T cells expressing hTERT WT but not in those expressing the catalytically inactive Q169A mutant (Figure 7B). As mentioned above, 293T cells contain functional endogenous telomerase. This allowed us to investigate whether hTERT Q169A inhibited the action of endogenous telomerase at telomeres. Bulk telomere length in cells stably expressing hTERT Q169A was similar to that of vector-only control cells (Figure 7B), although the vector-only cells have slightly longer telomeres upon infection. In both of these cell lines, telomeres do not shorten with increased passage number. Furthermore, neither the vector only cells nor the cells expressing Q169A underwent apoptosis and can be maintained in culture (data not shown), unlike other dominant negative alleles of hTERT [41], [42]. Collectively, these data indicate that hTERT Q169A does not function as a dominant negative protein in 293T cells.

**Figure 8:**
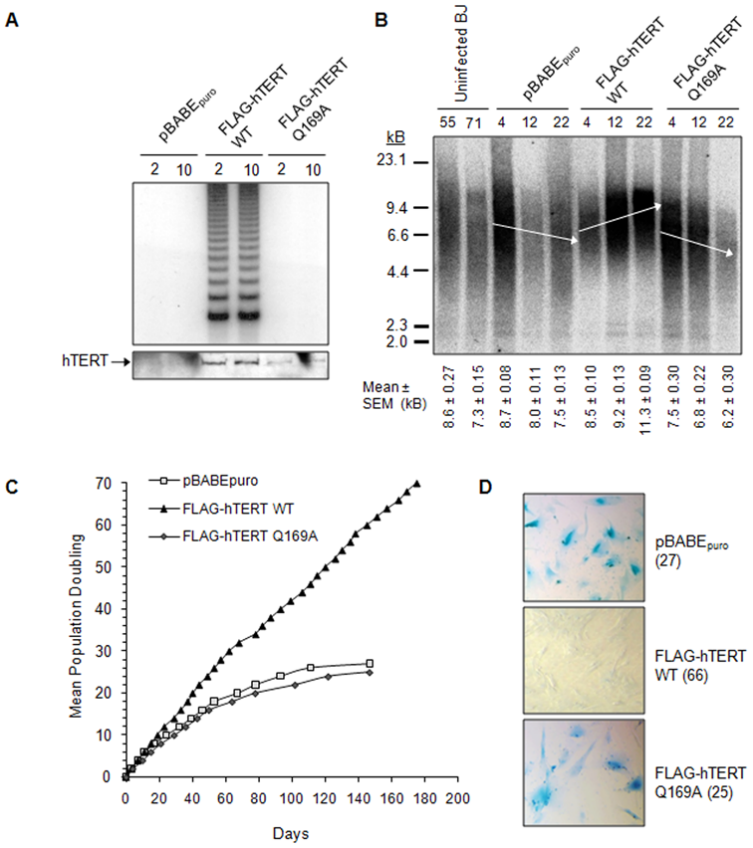
hTERT Q169 is required for telomerase activity, telomere length-maintenance, and hTERT-mediated immortalization in primary human cell lines. *A,* Extracts from BJ cells stably expressing pBABE_puro_, pBABE_puro_-FLAG-hTERT WT, or pBABE_puro_-FLAG-hTERT Q169A were immunoprecipitated with anti-FLAG antibodies. FLAG-hTERT was eluted from the beads by competition with excess 3 × FLAG peptide. 2 µL of eluate was tested for telomerase activity by the telomere repeat amplification protocol (top panel) and 35 µL was resolved by 8% SDS-PAGE and examined for hTERT expression by Western blotting with anti-hTERT antibodies (bottom panel). The mean population doubling is indicated above each lane. *B,* Bulk telomere length was measured using the terminal restriction fragment analysis. Mean telomere length and standard error of the mean (SEM) was calculated from at least three independent experiments and is indicated below each lane. The mean population doubling of each culture is indicated above the lane. Arrows are included for visual clarification and represent the approximate mean of each sample. *C,* Biological activity of hTERT Q169A was investigated by continuously passaging BJ cells to determine if the cultures entered replicative senescence or bypassed this proliferative blockade, as determined using growth curves. *D,* β-galactosidase activity at pH 6.0, a well-known marker of replicative senescence, was monitored using conventional staining techniques to detect blue-colored cells. Representative BJ cells stably expressing the empty vector or FLAG-hTERT Q169A exhibited blue staining that coincided with the cessation of proliferation. In contrast, cells stably expressing FLAG-hTERT stained negative for β-galactosidase activity (pH 6.0). The mean population doubling of each culture is indicated in parenthesis.

The cellular function of hTERT Q169A was also investigated in normal diploid fibroblasts (BJ cells). Telomerase negative BJ cells provide a strong cellular contrast to the telomerase positive 293T cells and thus, control for cell line-dependent phenotypes. Expression of hTERT WT, but not Q169A, conferred BJ cells with telomerase activity (Figure 8A). The lack of telomerase activity in immunoprecipitates containing hTERT Q169A was not due to abrogated protein expression (Figure 8A). Furthermore, stable expression of hTERT WT induced telomere elongation during early passages and telomere length maintenance during later passages (Figure 8B). These cultures completed approximately three times more mean population doublings (mpd) than the vector-only control cells, and did not exhibit senescence-associated β-galactosidase activity (Figures 8C and D, respectively) [43]. The BJ cells expressing hTERT Q169A exhibited defects in telomere length maintenance, exhausted their replicative potential after 25 mpd, and did not surmount replicative senescence (Figures 8B–D). Although the BJ cells expressing hTERT Q169 appear shorter at mpd 22 than the vector only control cells, the bulk telomere length of the control cells were slightly longer upon infection than in the hTERT Q169A cells due to the fact that these are polyclonal cell population However, the rates of telomere shortening in these cells are similar. Thus, mutation of hTERT Q169 inhibited human telomerase function in living cells.

## Discussion

### hTERT Q169 is essential for human telomerase activity

Important questions in telomerase biochemistry pertain to the mechanisms by which the enzyme recognizes and orientates telomeric ssDNA in the active site and catalyzes processive telomere synthesis. A long-standing notion is that telomerase has two modes of primer recognition: initial recognition of a telomere sequence and/or structure at the DNA 5′-end and subsequent identification of the 3′-terminus to prime the addition of telomeric nt [11]. Template-independent enzyme-DNA interactions are known as anchor site interactions and a subset of these are believed to confer telomerase with the property of repeat addition processivity. After synthesizing a complete telomeric repeat, Watson-Crick base pairs between the DNA 3′-end and RNA template are melted so that the nascent DNA 3′-end can be repositioned with the template for the next round of telomere synthesis. During this dynamic process, the telomerase anchor site(s) remains associated with upstream nt and prevents the enzyme from dissociating off the DNA primer.

The crystal structure of the *Tetrahymena* TEN domain revealed a ssDNA-binding groove on the surface that contained several phylogenetically-conserved residues, including the Gln residue investigated here [30], [33]. Alanine mutagenesis of tTERT Q168 significantly impaired telomerase activity without affecting repeat addition processivity and identified a role for this residue in ciliate telomerase anchor site interactions [30], [33]. The corresponding mutation in Est2p severely decreased yeast telomerase activity *in vivo* [31]. Here, we show for the first time that human telomerase reconstituted with hTERT Q169A, Q169D, or Q169N exhibits severe defects in DNA polymerization *in vitro* and *in vivo*, indicating that the invariant Gln residue has an evolutionarily-conserved role in telomerase function. Our study provides novel insight into the role of this residue by revealing its importance for the ability of telomerase to incorporate nucleotides at the second position of the RNA template. Importantly, the loss of telomerase activity *in vivo* was not due to nuclear exclusion or abrogated protein expression, although protein levels were reduced compared to hTERT WT. Interestingly, yeast strains expressing endogenous Est2p Q146A suffered severe growth defects and telomere attrition, and exhibited a reduction in Est2p protein levels [31], [44]. Thus, it appears that living cells do not tolerate high expression levels of TERT proteins harboring mutations of the invariant Gln residue.

In the absence of crystallographic data of hTERT, we can only speculate on the mechanism underlying the severity of the Q169D and Q169N mutations in relation to Q169A. As mentioned previously, the non-conservative Q169D substitution could cause electrostatic repulsion between the polyanionic DNA backbone and electronegative carboxylic acid side-chain. Our observation that the conservative Q169N does not rescue the catalytic phenotype indicates that both the length and the hydrogen-bonding potential of the side chain at position 169 are critical for the activity of human telomerase. The finding that hTERT Q169A reconstitutes weak telomerase activity is likely due to the fact that this is a neutral amino acid substitution. Although both Ala and Asn potentially eliminate important hydrogen-bonding interactions that would be normally fulfilled by Gln, the Q169N substitution is more detrimental to activity. One interpretation of this data is that the Asn substitution also introduces an unfavorable interaction whereby the shorter side chain prevents the amide group from satisfying its hydrogen-bonding potential.

Finally, we note that this residue appears to have evolved some species-specific functions. Alanine substitution of the corresponding residue in Est2p significantly impaired repeat addition processivity *in vitro* [31]. One intriguing explanation for these differences might be that telomerase uses different mechanisms to interact with different sequences of telomeric DNA. It is conceivable that ciliate and human telomerase use similar mechanisms to bind and synthesize telomeric DNA because of the high degree of telomere sequence similarity (TTGGGG and TTAGGG, respectively). In contrast, yeast telomeres are degenerate (*e.g.* G_2–3_(TG)_1–6_ in *S. cerevisiae*) [45] and different molecular mechanisms might be used for telomere length maintenance in this organism.

### Q169 alters the sensitivity of the hTERT N-terminus to proteolytic digestion

Telomerase is thought to contain template-distal and template-proximal anchor sites [11]. It has been speculated that a conformational change in the template-proximal anchor site is required for telomerase to transition into an elongation-competent complex [11]. Structure-function studies with *Tetrahymena* telomerase suggested that the ssDNA-binding groove on the surface of the TEN domain formed part of a template-proximal anchor site [30], [33]. Photo-cross-linking studies with tTERT and short telomeric ssDNA suggested that the TEN domain was displaced relative to the catalytic site during telomere synthesis. It was proposed that this movement was needed to reposition the template-proximal anchor site relative to the active site and correctly orientate the DNA primer or DNA-RNA heteroduplex in the active site during primer elongation [30], [33].

In this work, we investigated the structural significance of hTERT Q169 directly by using limited proteolysis to monitor the conformational flexibility of mutant proteins. Importantly, our results indicated that the overall conformation of hTERT Q169A, Q169D, and Q169N was intact. However, the NTE of the Q169 mutants was more sensitive to chymotrypsin proteolysis than hTERT WT. This is interpreted as evidence that Q169 is important for the conformation of the hTERT N-terminus. We believe that that a small fraction of the mutant proteins attained WT conformation because there was not a complete loss of the 20 kDa fragments. It is possible that these Q169 mutants are catalytically competent and thus, are responsible for the low levels of processive telomerase activity we observe *in vitro* (discussed below).

### hTERT Q169 mutants physically interact with hTR

TERT contains an essential and universally-conserved telomerase RNA-binding domain (TRBD) that makes extensive contacts with TR and represents the major TR-binding domain [46], [47]. It seemed unlikely that substitution of hTERT Q169 would disrupt hTERT-hTR interactions since this residue is not located in the TRBD. We confirmed here that hTERT Q169 is not involved in critical hTERT-hTR interactions. However, we cannot rule out the possibility that Q169 substitution causes small changes in hTR-binding that are masked in the context of the full length protein. Similarly, Q169 could regulate conformational changes in hTR or the RNP complex that are required for telomerase activity.

### Q169 mediates ssDNA-binding activity in hTERT

In this work we show that hTERT proteins containing Q169A, Q169D, or Q169N mutations retained telomere sequence-specific ssDNA-binding activity *in vitro*. However, when compared to hTERT WT, the Q169 mutants exhibited subtle differences in the relative strength of the interactions with telomeric ssDNA. In parallel, we investigated the DNA-binding properties of hTERT 1–300 variants containing the Q169 substitutions. hTERT 1–300 Q169A, Q169D, and Q169N also retained telomeric ssDNA-binding activity and demonstrated subtle differences in the relative strength of protein-DNA interactions when compared to 1–300 WT. While the presence of multiple DNA-binding sites in TERT complicates the interpretation of this data [28], [29], these studies clearly indicate that Q169 is involved in protein-telomeric DNA interactions, thereby identifying a novel residue in hTERT that regulates ssDNA-binding activity (discussed below). Future studies are required to determine Q169 has a direct or indirect effect on DNA-binding.

Interestingly, the hTERT Q169 mutations cause an increase in the relative strength of the interaction between the full length protein and short telomeric ssDNA primers whereas they decrease the interactions between hTERT 1–300 and these primers. We have previously identified multiple DNA-binding regions throughout the hTERT protein, including the N-terminus, RT domain, and C-terminus [28]. Similar results have now been reported for ciliate TERT [29]. Importantly, the DNA-binding regions appear to co-operate during telomeric ssDNA-binding *in vitro* and establish a dynamic ‘DNA-binding equilibrium’ in which the primer can interact with any or all of the DNA-binding domains [28], [29]. The data presented in Figures 5 and 6 suggests that the Q169 mutations have disrupted an important DNA-binding domain in the hTERT N-terminus thereby favoring an interaction with the remaining DNA-binding domains. Our observation that short ssDNA primers interact with the full length Q169 mutants to a greater extent than wild type hTERT implies that in the context of full length hTERT, Q169 negatively regulates the overall strength of the interaction with short telomeric primers. The apparent decrease in the strength of the interaction between bio-TELO30 and hTERT Q169D and Q169N, however, implies that Q169 may also be required to stabilize interactions with longer stretches of telomeric ssDNA. In contrast to full length hTERT, mutation of Q169 decreases the interaction between short telomeric primers and hTERT 1–300. One logical explanation for this decrease is that the 1–300 mutant lacks many of the previously identified DNA-binding regions in hTERT. Thus, Q169 has an important role in stabilizing the interactions that occur between the hTERT N-terminus and short telomeric ssDNA primers *in vitro*. Importantly, we have previously shown that the hTERT 1–300 fragment has a strong non-sequence-specific ssDNA-binding activity *in vitro* [28]. We interpreted this to mean that the hTERT N-terminus contains a strong ssDNA-binding activity but regions beyond the N-terminus are important for TTAGGG-specific ssDNA-binding. We show here that substitution of Q169 in the context of hTERT 1–300 causes a significant decrease in the ability of this fragment to bind ssDNA primers comprised of random or yeast telomeric ssDNA (Figure 6B). This observation identifies a previously unrecognized role for hTERT Q169 in regulating sequence-specific interactions between the protein's N-terminus and ssDNA primers *in vitro*. Our data supports previous studies that identified a potential role for Q168 in mediating ciliate telomerase's affinity for telomeric ssDNA [30], [33]. Collectively, these studies indicate that the Gln residue has an evolutionarily-conserved role in regulating TERT-telomeric ssDNA interactions (*i.e.* anchor site interactions).

### Conceptual model to describe the catalytic phenotype of hTERT Q169 mutants

Recently, Zaug *et al*. proposed a model for telomerase activity in which the catalytic cycle is regulated by a series of conformational changes [32]. It was suggested that telomerase initiates DNA synthesis when the TEN domain is in close proximity to the remainder of the RNP (‘closed state’). This conformation is regulated by an intramolecular protein-protein interaction between the TEN domain and an as yet unidentified residue [32]. Once the first round of telomere synthesis is complete, this interaction is broken and the TEN domain is displaced relative to the catalytic site, which relaxes the RNP into an ‘open state’. Displacement of the TEN domain repositions the template-proximal anchor site and template region, and restores base-pairing between the DNA 3′-end and template 5′-end. It is thought that the DNA 5′-end slides through the ssDNA-binding groove on the TEN domain, which signals the RNP to return to the closed state for the next round of telomere synthesis. This model invokes conformation changes within telomerase that are similar to those described for bacterial and eukaryotic RNA polymerases during the transition from transcription initiation to elongation (reviewed in [48], [49]). For example, the initial loading of promoter DNA onto RNA polymerase II triggers a series of structural changes that include the closure of an evolutionarily-conserved ‘clamp’ over the template and transcript. Clamp closure converts the enzyme from an open to a closed state that is required for transcription elongation. It is intriguing to speculate that the TEN domain could be involved in processes that are similar to the RNA polymerase clamp structure.

We interpret the increased protease-sensitivity of full length and 1–300 hTERT Q169 mutants as evidence that the N-terminus adopts a conformation that is more accessible to chymotrypsin than WT hTERT (*i.e.* greater conformational flexibility). We speculate that the mutants cannot easily convert between open and closed states (above), which alters critical telomerase-DNA interactions and causes severe catalytic defects. This seems particularly important for the ability of human telomerase to incorporate nucleotides at the second position of the hTR template. We suggest that the minimal activity observed with Q169 mutants *in vitro* stems from a small population of enzymes that correctly orientate the DNA primer and/or DNA-RNA hybrid in the active site to catalyze telomere synthesis. This is consistent with the fact that we do not observe a complete loss of the 20 kDa proteolytic fragment in our limited proteolysis experiments. According to the model proposed by Zaug *et al*., Q169 is not directly involved in repeat addition processivity [32], which is consistent with our observation that hTERT Q169A demonstrates normal repeat addition processivity. We propose that the mechanism by which Q169 regulates telomerase activity involves conformational changes that promote optimal enzyme-primer-substrate interactions and facilitate the efficient incorporation of nucleotides during the first round of telomere synthesis.

### Perspectives

In summary, we show that Q169 is a functionally critical residue in human telomerase and provide the first detailed evidence regarding the biochemical and cellular significance of this evolutionarily-conserved Gln residue in higher eukaryotes. The intimate relationship between telomerase and human disease underscores the importance for structure-function studies that elucidate regions in hTERT that could be amenable to therapeutic intervention. Our studies are necessary, but not sufficient, for a complete understanding of how the TEN domain contributes to telomerase structure, activity, and telomere length maintenance in higher eukaryotes. Importantly, since Q169 is essential for telomerase activity *in vivo*, this residue could represent a novel target for therapeutic intervention of telomerase activity in human diseases like cancer.

## Supporting Information

Figure S1 Q169 mediates hTERT conformation independently of hTR. RRL containing in vitro transcribed hTR and [35S]cysteine-labelled FLAG-hTERT WT, Q169A, Q169D, or Q169N was incubated with 0, 10, 100, or 1000 ng/mL chymotrypsin at 30°C for 2 min. 15 µL proteolysis reactions contained comparable amounts of [35S]cysteine-labelled hTERT. Reactions were terminated by the addition of SDS-PAGE loading buffer and boiling (5 min). Following digestion, products were resolved by 12% SDS-PAGE and visualized by autoradiography and phosphorimaging. Arrows indicate the proteolytic fragments that are diminished upon substitution of hTERT Q169.
(0.85 MB TIF)

Figure S2 Time-course proteolytic digestion of hTERT WT and Q169 mutants. RRL containing A, [35S]cysteine-labelled FLAG-hTERT WT, Q169A, Q169D, or Q169N or B, in vitro transcribed hTR and [35S]cysteine-labelled FLAG-hTERT WT, Q169A, Q169D, or Q169N was incubated with 500 ng/mL chymotrypsin at 30°C for 30 s, 1 min, 2 min, 5 min, 10 min, 15 min, 30 min, and 45 min. 15 µL proteolysis reactions contained approximately equivalent counts of [35S]cysteine-labelled hTERT. Reactions were terminated by the addition of SDS-PAGE loading buffer and boiling (5 min). Following digestion, products were resolved by 12% SDS-PAGE and visualized by autoradiography and phosphorimaging. Arrows indicate the proteolytic fragments that are reduced upon substitution of hTERT Q169.
(1.31 MB TIF)

Figure S3 Time-course proteolytic digestion of 1–300 hTERT WT and Q169 mutants. RRL containing A, [35S]cysteine-labelled 1–300 FLAG-hTERT WT, Q169A, Q169D, or Q169N or B, in vitro transcribed hTR and [35S]cysteine-labelled 1–300 FLAG-hTERT WT, Q169A, Q169D, or Q169N as incubated at 30°C with 500 ng/mL chymotrypsin for the indicated time (30 s, 1 min, 2 min, 5 min, 10 min, 15 min, 30 min, and 45 min), terminated by the addition of SDS-PAGE loading buffer and boiling (5 min), and resolved by 12% SDS-PAGE. 15 µL proteolysis reactions contained comparable counts of [35S]cysteine-labelled hTERT. Following digestion, products were resolved by 12% SDS-PAGE and visualized by autoradiography and phosphorimaging. Arrows indicate the proteolytic fragments that are reduced upon substitution of Q169 in 1–300 hTERT.
(1.05 MB TIF)
