# Supplementary figures and images for: Human Telomerase Reverse Transcriptase (hTERT) Q169 Is Essential for Telomerase Function In Vitro and In Vivo
Source: PLoS One. 2009 Sep 24;4(9):e7176. doi: 10.1371/journal.pone.0007176 (PMC2744565; doi:10.1371/journal.pone.0007176)

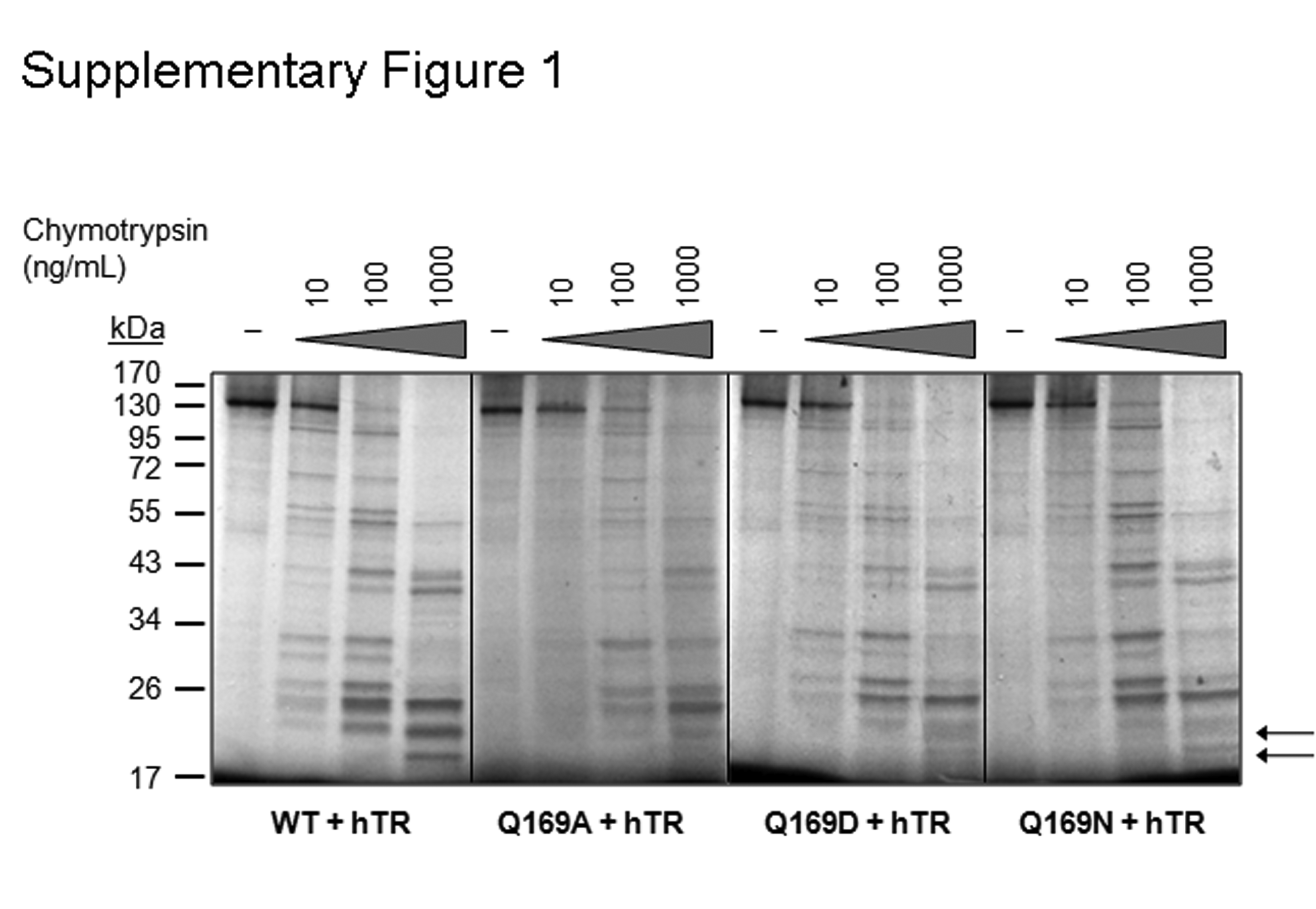

Supplement: Figure S1 — Q169 mediates hTERT conformation independently of hTR. RRL containing in vitro transcribed hTR and [35S]cysteine-labelled FLAG-hTERT WT, Q169A, Q169D, or Q169N was incubated with 0, 10, 100, or 1000 ng/mL chymotrypsin at 30°C for 2 min. 15 µL proteolysis reactions contained comparable amounts of [35S]cysteine-labelled hTERT. Reactions were terminated by the addition of SDS-PAGE loading buffer and boiling (5 min). Following digestion, products were resolved by 12% SDS-PAGE and visualized by autoradiography and phosphorimaging. Arrows indicate the proteolytic fragments that are diminished upon substitution of hTERT Q169. (0.85 MB TIF) [file pone.0007176.s001.tif]

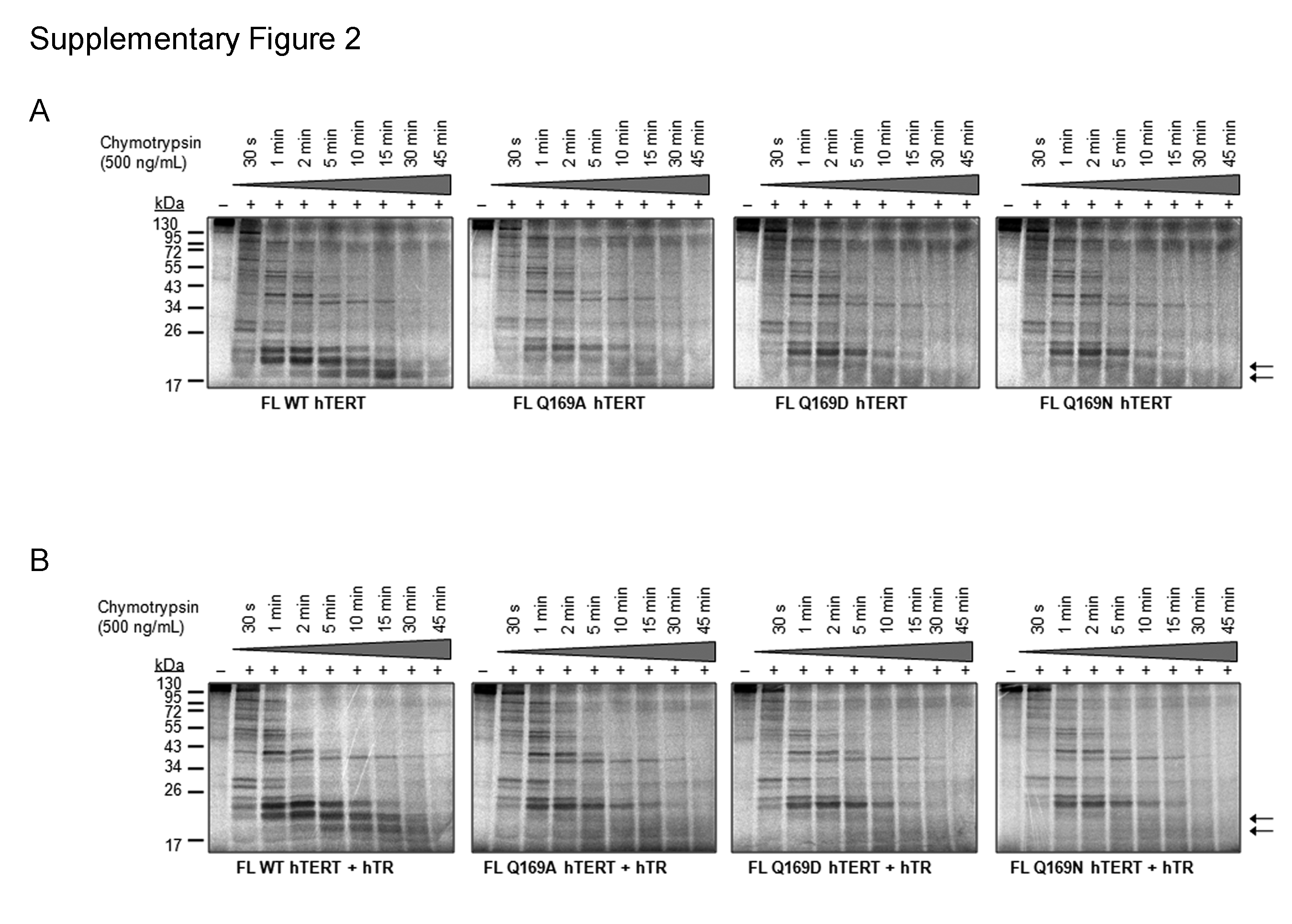

Supplement: Figure S2 — Time-course proteolytic digestion of hTERT WT and Q169 mutants. RRL containing A, [35S]cysteine-labelled FLAG-hTERT WT, Q169A, Q169D, or Q169N or B, in vitro transcribed hTR and [35S]cysteine-labelled FLAG-hTERT WT, Q169A, Q169D, or Q169N was incubated with 500 ng/mL chymotrypsin at 30°C for 30 s, 1 min, 2 min, 5 min, 10 min, 15 min, 30 min, and 45 min. 15 µL proteolysis reactions contained approximately equivalent counts of [35S]cysteine-labelled hTERT. Reactions were terminated by the addition of SDS-PAGE loading buffer and boiling (5 min). Following digestion, products were resolved by 12% SDS-PAGE and visualized by autoradiography and phosphorimaging. Arrows indicate the proteolytic fragments that are reduced upon substitution of hTERT Q169. (1.31 MB TIF) [file pone.0007176.s002.tif]

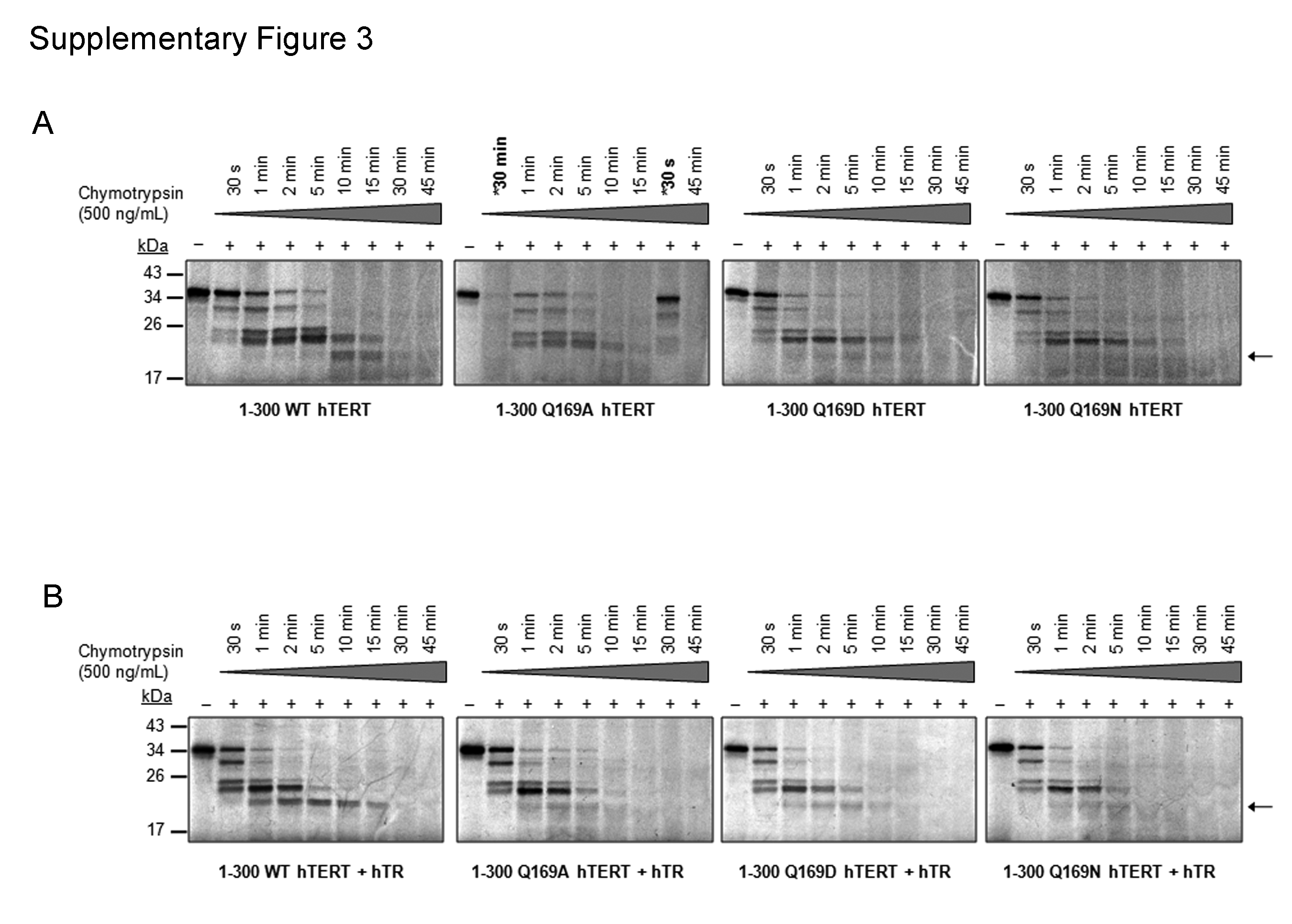

Supplement: Figure S3 — Time-course proteolytic digestion of 1–300 hTERT WT and Q169 mutants. RRL containing A, [35S]cysteine-labelled 1–300 FLAG-hTERT WT, Q169A, Q169D, or Q169N or B, in vitro transcribed hTR and [35S]cysteine-labelled 1–300 FLAG-hTERT WT, Q169A, Q169D, or Q169N as incubated at 30°C with 500 ng/mL chymotrypsin for the indicated time (30 s, 1 min, 2 min, 5 min, 10 min, 15 min, 30 min, and 45 min), terminated by the addition of SDS-PAGE loading buffer and boiling (5 min), and resolved by 12% SDS-PAGE. 15 µL proteolysis reactions contained comparable counts of [35S]cysteine-labelled hTERT. Following digestion, products were resolved by 12% SDS-PAGE and visualized by autoradiography and phosphorimaging. Arrows indicate the proteolytic fragments that are reduced upon substitution of Q169 in 1–300 hTERT. (1.05 MB TIF) [file pone.0007176.s003.tif]
